# Clinical Manifestations of Systemic Lupus Erythematosus in a Tertiary Center in Saudi Arabia

**DOI:** 10.7759/cureus.41215

**Published:** 2023-06-30

**Authors:** Mohammed AlOmair, Hanan AlMalki, Mohammed AlShahrani, Hadeel Mushait, Maraam Al Qout, Talal Alshehri, Reem AlAlyani, Amjd Algarni, Yazan Almaker, Elaf Madkli

**Affiliations:** 1 Rheumatology, Aseer Central Hospital, Abha, SAU; 2 Rheumatology, King Khalid University Medical City, Abha, SAU; 3 College of Medicine, King Khalid University, Abha, SAU; 4 College of Medicine, Jazan University, Jazan, SAU

**Keywords:** systemic lupus erythematosus, sle, antinuclear antibodies (ana), lupus, clinical manifestations, kingdom of saudi arabia (ksa)

## Abstract

Introduction

Systemic lupus erythematosus (SLE) is a chronic autoimmune disease with multisystemic involvement. The clinical presentation and immunological findings of SLE patients from different regions in Saudi Arabia have been studied. There have been no studies on the clinical manifestations of SLE in patients in Saudi Arabia's southern region. This article aims to explore the clinical manifestations of SLE in a tertiary center in the southern region of Saudi Arabia.

Methods

A retrospective study was carried out on 108 SLE patients who were seen in the rheumatology clinic at Aseer Central Hospital over six months from January 2022 to June 2022. Patients’ demographics, clinical and serological characteristics, and therapeutic data were reviewed.

Results

The male-to-female ratio was 1:12.5, with a mean age at presentation of 28.6 ± 10 years. The mean disease duration was 9.06 ± 5.96 years. Mucocutaneous and musculoskeletal manifestations were the most common, accounting for 76% and 57% of all cases, respectively. Neuropsychiatric involvement and lupus nephritis were present in 29% and 31% of patients, respectively. The hematological abnormalities that were present included anemia (60%), leukopenia (37%), and thrombocytopenia (15%). Antinuclear antibody (ANA) was detected in 100%, anti-double-stranded DNA (anti-dsDNA) antibody in 55%, anti-Smith antibody in 13%, and hypocomplementemia in 52% of patients. Hydroxychloroquine was received by 98% and oral steroids by 41% of the patients. Other drugs include azathioprine (23%), mycophenolate mofetil (15%), methotrexate (23%), belimumab (9%), cyclophosphamide (10%), and rituximab (6%).

Conclusion

The main clinical features of our patients were in parallel with previous studies in Saudi Arabia as well as in Arab countries. We found a lower prevalence of lupus nephritis, serositis, and anti-dsDNA antibody. Further multicenter studies are required to investigate the long-term outcome and survival of SLE patients.

## Introduction

Systemic lupus erythematosus (SLE) is a chronic autoimmune disease with multisystemic involvement. It is known to have a worldwide distribution, female predominance, and poorly known etiology. The clinical presentation of SLE is highly variable, ranging from mild skin and joint manifestations to severe major organ involvement [1]. The disease course is characterized by chronic and acute attacks and periods of remission [2]. Although it is a multisystemic disease, renal and neurological manifestations are the most serious complications [3]. Fatigue, fever, and weight loss are constitutional symptoms that are typically present in all SLE patients [4].

There are significant disparities in the incidence and prevalence of SLE around the world. In a systematic review of epidemiological studies that reported SLE incidence rates, the highest incidence was reported in North America (23.2/100,000 person-years), and the lowest incidences were reported in Africa and Ukraine (0.3/100,000 person-years for each) [5]. The prevalence of SLE in Saudi Arabia was estimated to be 19.28 per 100,000 population [6].

Genetic contributions have been found to be associated with SLE, where patients of Hispanic, African American, and Asian descent had an early onset of the disease and a severe presentation [7]. Studies on SLE patients from Arabic countries have revealed a similar age of onset, female predominance, and many similarities in clinical presentation and immunological findings [8].

To date, no studies have investigated the clinical presentation of SLE among patients in the southern region of Saudi Arabia. For that reason, the aim of this study was to determine the clinical manifestations of SLE in a tertiary center in Saudi Arabia.

## Materials and methods

A retrospective study was conducted on SLE patients who were seen in the rheumatology outpatient clinic at Aseer Central Hospital over six months from January 2022 to June 2022. All patients were aged 18 years or older and satisfied at least four of the Systemic Lupus International Collaborating Clinics (SLICC) classification criteria for the classification of SLE [9]. Patients who were lost to follow-up or had incomplete clinical data were excluded.

Medical records of the patients were reviewed for patient demographics (sex, current age, age at diagnosis, comorbidities, and duration of SLE). The main SLE manifestations evaluated in this study were constitutional symptoms, musculoskeletal, mucocutaneous involvement, cardiopulmonary involvement, Raynaud phenomenon, thromboembolic disease, gastrointestinal involvement, neuropsychiatric involvement, and lupus nephritis. Laboratory data include complete blood count (CBC), creatinine, inflammatory markers, urine analysis, proteinuria, and the immunological profile, including antinuclear antibody (ANA), anti-double-stranded DNA (anti-dsDNA) antibody, anti-Smith antibody, rheumatoid factor (RF), complement (C3 and C4), anti-Sjogren's syndrome-related antigen A (anti-SS-A) and anti-Sjogren's syndrome-related antigen B (anti-SS-B) antibodies, and antiphospholipid antibodies. In the case of lupus nephritis, the results of the renal biopsy were documented. Renal biopsies were evaluated according to the International Society of Nephrology/Renal Pathology Society classification [10]. Treatment modalities such as steroids and immunosuppressive drugs were reviewed.

Ethical approval was obtained from the Ministry of Health, Asser Regional Committee for Research Ethics. As there was no direct influence on the participants, informed patient consent was not required.

Data analysis

The data were analyzed using the Statistical Package for the Social Sciences (SPSS) database program (IBM Corp., Armonk, NY). For continuous variables, descriptive statistics were performed, including the number of observations, mean, minimum, and maximum. For categorical variables, counts and percentages were used. The mean with standard deviation and median with range were used to describe scale data according to the shape of the distribution.

## Results

During the period from January 2022 to June 2022, 108 patients fulfilling at least four of the SLICC criteria for SLE were enrolled in this study. Table 1 illustrates the demographic data of the patients.

**Table 1:** Demographic characteristics of our patients

Variable	All patients	
	N	%
Age (years)		
<50	94	87.0%
≥51	14	13.0%
Age at disease onset (years)		
<16	11	10.2%
17-50	93	86.1%
≥51	4	3.7%
Gender		
Male	8	7.4%
Female	100	92.6%
Male-to-female ratio = 1:12.5		
Comorbidities		
None	59	54.6%
Antiphospholipid syndrome	22	20.4%
Thyroid disease	22	20.3%
Hypertension	19	17.6%
Diabetes mellitus	10	9.3%
Others	16	14.8%
Asthma	4	3.7%
Gastroesophageal reflux disease	4	3.7%
Rheumatic heart disease	3	2.8%
Chronic kidney disease	3	2.8%
Celiac disease	2	1.9%

The study comprised 100 females (92.6%) and eight males (7.4%), for a male-to-female ratio of 1:12.5. The patients' mean age at disease onset was 28.6 ± 10 years, while the duration of disease was 9.06 ± 5.96 years. Comorbid illnesses were found in 45.4% of SLE patients. Thyroid gland disorders, hypertension, and diabetes mellitus were prevalent in 20%, 18%, and 9% of patients, respectively. A total of 22 patients (20%) had secondary antiphospholipid syndrome. Table 2 summarizes the clinical manifestations of SLE in the study group.

**Table 2:** The clinical manifestations of SLE patients SLE: systemic lupus erythematosus; LN: lupus nephritis.

Variable	All patients	
	N	%
Constitutional symptoms	108	100%
Fever	20	18.6%
Fatigue	70	64.8%
Weight change	18	16.6%
Mucocutaneous involvement	82	75.9%
Acute cutaneous lupus	42	38.9%
Subacute/chronic cutaneous lupus	20	18.5%
Oral/nasal ulcers	33	30.6%
Hair loss	29	26.9%
Cutaneous vasculitis	8	7.4%
Arthritis	62	57.4%
Lupus nephritis	34	31.4%
LN class I or II	8	23.5%
LN class III	3	8.8%
LN class IV	15	44.1%
LN class V	2	5.8%
Unknown	6	17.6%
Neuropsychiatric involvement	31	28.7%
Seizures	9	8.3%
Depression	9	8.3%
Stroke	9	8.3%
Cerebritis	4	3.7%
Thromboembolic disease	22	20.4%
Venous	18	16.7%
Arterial	10	9.3%
Pregnancy complications	12	11.1%
Lymph node enlargement	10	9.3%
Cardiopulmonary involvement	9	8.33%
Pleuritis	3	2.8%
Pulmonary hypertension	2	1.9%
Alveolar hemorrhage	2	1.9%
Myocarditis	2	1.9%
Raynaud phenomenon	8	7.4%
Gastrointestinal involvement	4	3.7%

Mucocutaneous and musculoskeletal manifestations were the most frequently observed, at 76% and 57%, respectively. Constitutional symptoms, though nonspecific, were reported by almost all of the patients. Lupus nephritis was present in 31% (34 patients). The results of the renal biopsy revealed that 44% of the cases had class IV histological findings, 23.5% had class I or II, 8.8% had class III, and 6% had class V histological findings. The renal biopsy could not be performed on six patients because they were critically ill. Regarding cardiopulmonary involvement, 7% of patients had serositis, and myocarditis and alveolar hemorrhage occurred in 2% of patients for each. Neuropsychiatric involvement was noted in 29% of patients (31 patients). Anemia, leukopenia, and thrombocytopenia rates were 60%, 37%, and 15%, respectively, among our patients. Table 3 shows the laboratory findings and immunological parameters of the patients.

**Table 3:** The laboratory findings and immunological parameters of our patients CRP: C-reactive protein; ESR: erythrocyte sedimentation rate; ANA: antinuclear antibody; dsDNA: double-stranded DNA; B2GP1: beta-2 glycoprotein 1; RF: rheumatoid factor; anti-SS-A: anti-Sjogren's syndrome-related antigen A; anti-SS-B: anti-Sjogren's syndrome-related antigen B.

Variable	All patients		
	N	%	
Anemia	65	60.19%	
Leukopenia	40	37.04%	
Thrombocytopenia	16	14.81%	
High creatinine	11	10.19%	
Proteinuria	34	31.48%	
High CRP	67	62.04%	
High ESR	57	52.78%	
ANA	104	96.2%	
Anti-dsDNA	59	54.6%	
Anti-Smith	14	12.96%	
Low complement	56		51.85%
Anti-SSA	9		8.33%
Anti-SSB	9		8.33%
Anti-B2GP1 IgG	18		16.67%
Anti-B2GP1 IgM	11		10.19%
Anti-cardiolipin IgG	18		16.67%
Anti cardiolipin IgM	11		10.19%
Lupus anticoagulant	4		3.7%
RF	9		8.33%

The majority of patients (96.2%) had a positive ANA titer. The titer was as low as 1:40 in four patients with cutaneous symptoms but no involvement of internal organs. Of 101 patients who underwent anti-dsDNA antibody, 59 (55%) tested positive. Anti-Smith antibody was tested in half of the studied patients, and one-third of them had a positive result. Low complements levels were seen in 56% of patients.

At the time of the last follow-up, 106 (98%) and 45 (41%) of patients were taking hydroxychloroquine and steroids, respectively. Intravenous cyclophosphamide was received by 11 (10%) and rituximab by seven (6%) of the patients. Vitamin D and calcium were given to 99 (90%) of the patients. The other prescribed immunosuppressive medications are detailed in Table 4.

**Table 4:** Medications taken by patients with systemic lupus erythematosus

Variable	All patients	
	N	%
Hydroxychloroquine	106	98.15%
Methotrexate	25	23.15%
Azathioprine	25	23.15%
Mycophenolate mofetil	16	14.81%
Tacrolimus	3	2.77%
Belimumab	10	9.26%
Rituximab	7	6.48%
Cyclophosphamide	11	10.19%
Steroid		
Current steroid use	45	41.67%
≤5 mg	33	73.33%
7.5-10 mg	7	15.5%
10-20 mg	5	11.11%
History of pulse steroid	24	22.22%
Vitamin D	102	94.4%
Calcium	72	66.7%

## Discussion

This is the first review of the clinical manifestations of SLE in the southern region of Saudi Arabia. SLE predominantly affects women of reproductive age, and the female-to-male ratios vary significantly across regions of the world. Our finding reveals a female-to-male ratio of 12.5:1, in line with that reported in Arab countries. Our reported female-to-male ratio was higher than in Europe, Africa, and China, but lower than in Hispanics [11-13]. The pediatric-onset SLE frequency was 10%, which is consistent with that reported in non-Caucasian patients [14]. Thyroid gland disorders were the most prevalent comorbidities in our patients, which is consistent with numerous studies that have shown a significant association between SLE and thyroid gland disorders [15,16]. In a Saudi study, thyroid gland dysfunction was found in 17% of SLE patients [17].

Table 5 summarizes the various clinical and serologic features that were previously published in Saudi Arabia [18-21].

**Table 5:** Comparison of clinical characteristics of SLE patients from various Saudi Arabia regions SLE: systemic lupus erythematosus; ESR: erythrocyte sedimentation rate; ANA: antinuclear antibody; anti-dsDNA: anti-double stranded DNA; LA: lupus anticoagulant; ACL: anticardiolipin; B2GP1: beta 2 glycoprotein 1; RF: rheumatoid factor; VTE: venous thromboembolism.

	Our study (108 patients)	Riyadh (642 patients) (2009)	Jeddah (65 patients) (2002)	Tabuk (73 patients) (2016)	Al-Ahsa (45 patients) (2013)
Constitutional symptoms					
Fever	18.6%	30.6%,	9.2%		
Fatigue	64.8%	42.5%,	9.2%		
Weight loss	16.6%	23.1%			
Lymph node enlargement	9%	20.0%			
Arthralgia/arthritis	57.4%	80.4%	60%	71%	91.3%
Mucocutaneous manifestations					
Malar rash	39%	47.9%	20%	61.6%,	67.4%
Photosensitivity	39%		24.6%	63%	47.8%
Discoid rash	18.5%	17.6%		15.1%	13%
Alopecia	26.9%	47.6%	21.5%		65.2%
Oral ulcers	31%	39.1%		47.9%	
Raynaud phenomenon	7.4%	8.7%			15.2%
Serositis	7%	27%			
Cardiac involvement					
Myocarditis	2%				13.0%
Pericarditis		20.7%	1.5%		10.9%
Pulmonary involvement					
Pleuritis	3%	15.8%	4.6%		6.5%
Pneumonitis		1.6%	1.5%		
Interstitial lung disease		4.5%			4.4%
Hepatosplenomegaly	4%	6.1%	7.6%		
Neuropsychiatric involvement	28.7%	27.6%	26%	35.6%	28.3%
Lupus nephritis	31.4%	47.9%	55.4%	44.1%	58.7%
Anemia	60.1%	63%	34%	64.4%	47.8%
Leukopenia	37%	30.1%	37%	54.8%	58.7%
Thrombocytopenia	14.8%	10.9%	1.5%	21.9%	32.6%
High ESR	52.7%	54.6	37%	93.7%	
ANA	96.2%	99.7%	87%	98.6%	95.7%
Anti-dsDNA antibody	54.6%	80.1	92%	100%	82.6%
Anti-Smith antibody	12.9%	41.6%	69%	26%	30.4%
Hypocomplementemia	51.8%	54.4%	67%		89.1%
LA	3.7%	27%	15%	3.9%	17.4%
ACL antibody	16.6%	49.7%		13.7%	17.4%
B2GP1 antibody	16.6%			9.9%	
Anti-Ro	8.3%	53.1%		77.8%	82.6%
Anti-La	8.3%	26.6%			82.6%
RF	8.3%	23%	15%		
VTE	16.7%	10.7%	12%	15.1%	15.2%
Abortions	11.1%		3%		

Acute cutaneous lupus, particularly malar rash and photosensitivity, was present in 39% of our patients. In comparison, photosensitivity affected up to 91.5% of Egyptians and 16% of Lebanese [8]. Discoid rash was documented in 18.5% of our patients. It was uncommon in Sudan and Kuwait affecting 2.4% and 7%, respectively [8]. Photosensitivity and malar rash are seen more frequently in European patients, whereas discoid is more common in Blacks [12].

Arthropathy was a common symptom (57% of the patients) in this study, and this result is consistent with findings reported from Egypt (52%) and Oman (47%). However, arthropathy ranged from 84% (Tunisia, UAE, Jordan, and Kuwait) to 95% (Lebanon) [8]. The present study had the lowest incidence of serositis (7%) compared to studies in Egypt (44%) and Tunisia (47%) [8]. Hispanics have the highest incidence of serositis, reaching up to 64% in a multiethnic cohort [11].

Lupus nephritis was found in 31.4% of our patients, which is lower than the previous studies in Saudi Arabia and Arab countries. Positive anti-dsDNA antibodies are well known to be strongly associated with lupus nephritis [22], which could explain the lower prevalence of anti-dsDNA antibodies in our patients.

When compared to Europeans, Africans and Hispanic descents are associated with more prevalent renal involvement [23]. In the series of Lee et al., up to 69.3% of Chinese patients had renal disease [24].

Neuropsychiatric manifestations affected a third of our patients. In two studies conducted at Aseer Central Hospital, 23.5% and 52% of patients had at least one neurological or psychiatric disorder, respectively [25,26]. In an analysis of 3,273 Arab patients, there was no significant difference in the frequency of neuropsychiatric manifestations when compared to the Euro-lupus cohort [8].

When compared to Arab countries, the frequency of serositis was similar in our study; one was from Yemen (9.4%), with Egypt and Tunisia having the highest rates (44 and 47%, respectively) [8]. In terms of hematological abnormalities, our results were comparable to other studies in Arab patients. In the California Lupus Surveillance Project, hematological manifestations were found to be more common among Blacks, Asians, and Hispanics [27].

The patients who had negative ANA had cutaneous lupus without other organ involvement. Anti-dsDNA antibody was positive in half of the patients in parallel to the Iraq, Lebanon, Kuwait, and Sudan populations. Anti-dsDNA antibody was found to be lower in Arabs than in Europeans [8]. When compared to previously published studies in Saudi Arabia, we have the lowest prevalence of anti-Smith and anti-dsDNA antibodies. It can be explained by the fact that in Saudi and Arabic studies, we have the lowest prevalence of lupus nephritis. Concerning the anti-Smith antibody, it was done in half of our patients, and it could be higher than documented.

Although serological tests were not done in all patients and it could be higher than documented, the results of anti-SS-A, anti-SS-B, anti-Smith antibodies, and anti-cardiolipin IgG and IgM were similar to Arabs from Oman, United Arab Emirates, and Tunisia [8]. In one study, the Afro-Caribbean population had the highest prevalence of anti-Smith, anti-SS-A, and anti-SS-B antibodies compared to Europeans and Asians [28]. The prevalence of antiphospholipid antibodies varies greatly among different populations [29].

Due to the variety of SLE clinical manifestations based on race and geographical area, our goal is to provide information about SLE in our population and compare it to Arabs and other ethnicities. We presented the first study reviewing clinical and serological manifestations of lupus in the southern region of Saudi Arabia, but it has some limitations. Firstly, it was conducted in a single center. Given that this was a retrospective study, it is not an appropriate study design to assess disease activity and outcomes. The results of serological tests may be higher than documented, which is explained by the fact that some patients were diagnosed elsewhere and the serological tests were not repeated in all of them.

## Conclusions

Our findings revealed that the main clinical disease spectrum was comparable to previous studies in Saudi Arabia as well as in Arab countries. We found lower frequencies in lupus nephritis, serositis, and anti-dsDNA antibody. More multicenter studies in Saudi Arabia are needed to investigate SLE complications and outcomes.
